# Liposome-based Expression of the PIEZO1 Sensor GenEPi in Hippocampal Neurons in Organotypic Slices

**DOI:** 10.21769/BioProtoc.5780

**Published:** 2026-08-05

**Authors:** Anya Bhavnani, Olga Kopach

**Affiliations:** 1 School of Health & Medical Sciences, City St George’s University of London, Cranmer Terrace, London, UK; 2 Queen Square Institute of Neurology, University College London, London, UK

**Keywords:** Organotypic hippocampal slices, PIEZO1, Optical sensor, Pyramidal neurons, Lipofectamine transfection, Large DNA constructs, Fluorescence imaging

## Abstract

Expressing large DNA constructs in the native three-dimensional brain microenvironment remains technically challenging. Although viral vectors provide high transduction efficiency and cell-type selectivity, their genetic payload capacity is limited. Various non-viral approaches have been used in brain tissue, but they may compromise tissue viability or require specialised equipment, such as biolistic delivery or electroporation. We present an adapted protocol for delivering the large DNA vector encoding the optical PIEZO1 sensor GenEPi into brain tissue to enable sensor expression in pyramidal neurons. By applying DNA–Lipofectamine liposomes directly to the slice surface, we achieved efficient, minimally invasive transfection of pyramidal neurons in the CA1 and CA3 regions of organotypic hippocampal slices. PIEZO1 sensor expression was detectable as early as 7 days after transfection, increased with longer tissue maintenance, and was sustained for 3–4 weeks in vitro. This protocol describes a cost-effective, non-invasive approach that preserves cell viability and enables investigation of PIEZO1-mediated mechanotransduction in a native brain microenvironment.

Key features

• DNA–Lipofectamine liposomes are applied directly to slice surface, enabling efficient transfection of superficial hippocampal neurons, important for imaging experiments performed using upright microscope systems.

• The protocol provides a cost-effective gene delivery approach that requires only small volumes of DNA and transfection reagent.

• Robust expression of the PIEZO1 sensor GenEPi is achieved within a relatively short time (approximately 1 week after transfection).

• The method is compatible with long-term tissue maintenance, with neuronal viability and GenEPi expression maintained for up to 3–4 weeks after transfection.

## Graphical overview


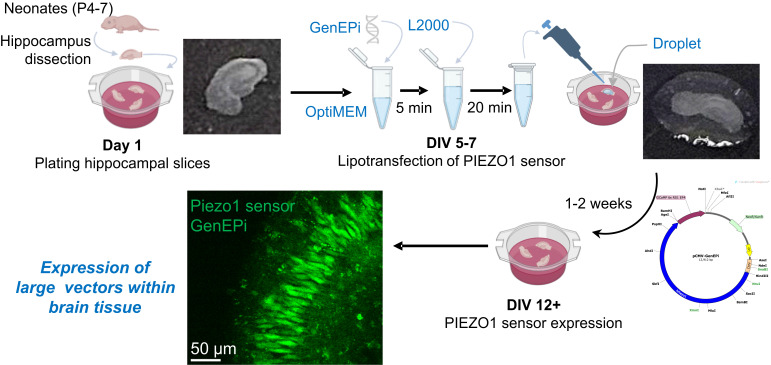


**Schematic illustration of lipotransfection of the PIEZO1 sensor GenEPi, a large DNA construct, in organotypic hippocampal slices.** A freshly prepared droplet of DNA–Lipofectamine 2000 complex is applied to the surface of individual hippocampal slices approximately 1 week after plating. This approach enables expression of the PIEZO1 sensor GenEPi as early as 1 week post-transfection, corresponding to approximately 12 days in vitro (DIV).

## Background

Genetically encoded tools have become essential for probing protein function in complex biological systems. However, many receptors and ion channels have multi-domain configurations encoded by genes whose expression constructs exceed the packaging capacity of commonly used viral vectors. As a result, expressing large DNA constructs remains a major technical challenge, and gene delivery methods are often limited in their ability to achieve robust and sustained expression in brain cell types. Several delivery approaches have been developed for this purpose, including viral vectors, biolistics (gene gun), electroporation, and lipotransfection [1]. Viral vectors, such as adeno-associated viruses, offer stable long-term expression with high transduction efficiency and relatively low cytotoxicity [2], yet their packaging capacity is limited to ~5 kb, restricting their use for larger constructs [3]. By contrast, non-viral methods can support the delivery of larger constructs and may enable access to deeper tissue layers, but they are often more invasive, may disrupt tissue integrity, and typically require specialised equipment [1,4]. Inefficient delivery, often associated with low expression levels and reagent-induced toxicity, therefore restricts the ability to achieve robust and sustained expression of large constructs when studying large, multi-domain channels.

This limitation is critically relevant for mechanosensitive ion channels of the PIEZO family, which form large complexes within the membrane. PIEZO1 represents a large homo-trimeric protein [5] and is a challenging target for genetic manipulations, particularly because of the technical constraints of current gene delivery approaches. Existing genetically encoded optical PIEZO1 sensors, such as GenEPi and Ca^2+^-sensitive HaloTag ligands, have been designed to report PIEZO1 conformational changes by detecting mechanosensitive Ca^2+^ influx [6,7]. While these sensors have been tested in cell models and in zebrafish, there are no reports of their successful implementation in brain neurons, either in primary cultures or in organised brain tissue preparations. The PIEZO1-specific sensor GenEPi has been validated in HEK293T, HFF, and HeLa cells, cardiomyocytes, and zebrafish [6]; however, its large vector size (12,912 bp) and structural complexity further exacerbate the limitations of classical viral and non-viral delivery methods. Despite the growing interest in PIEZO1-mediated mechanosensation within the brain, approaches that enable successful expression of the PIEZO1 sensor in brain cell types remain to be developed.

Organotypic hippocampal slices provide a powerful model for studying neuronal function in a preserved 3D environment. Unlike monolayer cultures, organotypic slices retain the layered architecture, synaptic circuitry, and cell-type diversity of the hippocampus, together with native cell–cell interactions [1,4,8], while offering experimental access comparable to in vivo settings. This combination of structure and accessibility makes organotypic slices particularly well-suited for investigating channels and receptors that operate within a native environment, such as mechanosensitive PIEZO1 channels. When combined with genetic manipulation, organotypic brain slices provide a valuable platform for studying protein and/or channel function within a native tissue structure that is essential for mechanobiology in a physiologically relevant context.

Here, we describe a modified, minimally invasive protocol for expressing large DNA constructs in organotypic hippocampal slices. Lipid-based transfection, such as Lipofectamine 2000–mediated delivery, is a well-established non-viral method for DNA transfer that is relatively simple, cost-effective, and less invasive than mechanical delivery techniques [9,10]. Lipid–nucleic acid complexes enter cells primarily via endocytosis; however, in thick tissue, diffusion barriers within the extracellular matrix can limit reagent penetration and reduce transfection efficiency. Existing protocols often rely on introducing large volumes of DNA–Lipofectamine complexes into the culture at very early stages, with short incubation periods [1,11]. This may dilute the transfection reagent, reduce targeting efficiency, or compromise tissue integrity. We provide a step-by-step description for applying DNA–Lipofectamine 2000 complexes directly onto the tissue surface, thereby maximising reagent access to the superficial layers while minimising physical stress to the slices. Using this approach, we enable efficient expression of the genetically encoded PIEZO1 sensor GenEPi in hippocampal pyramidal neurons as early as 7 days post-transfection, with expression maintained for 3–4 weeks. The protocol combines cost-effectiveness, straightforward implementation, and compatibility with standard imaging setups, making it suitable for functional studies of PIEZO1-mediated mechanotransduction that require rapid and sustained expression of large DNA constructs in intact 3D brain tissue.

## Materials and reagents

**Biological materials**

1. Sprague-Dawley rats (*Rattus norvegicus*) from Charles River Laboratories, strain code: 001 (neonates, P4–P6, of both sexes)

2. Alternatively, C57BL/6 J mice (*Mus musculus*) from Charles River Laboratories, strain code: 632 (neonates, P4–P7, of both sexes)

3. pCMV-GenEPi (Addgene plasmid #140236; http://n2t.net/addgene:140236; RRID: Addgene_140236)

*Note: For long-term storage, plasmid DNA was maintained at -20 °C with minimal freeze/thaw cycles. We successfully expressed GenEPi in organotypic slices using DNA concentrations of 400–1,000 ng/μL. DNA purity was within the range of A_260_/A_280_ ratio 1.8–2.0 and A_260_/A_230_ ratio 2.0–2.2.*

**Reagents**

1. Glucose (Sigma-Aldrich, catalog number: G8270)

2. Sodium chloride (Sigma-Aldrich, catalog number: S9888)

3. Sodium bicarbonate (Sigma-Aldrich, catalog number: S0751)

4. Sodium monophosphate (NaH_2_PO_4_) (Sigma-Aldrich, catalog number: S6040)

5. Potassium chloride (Sigma-Aldrich, catalog number: P9333)

6. Magnesium chloride hexahydrate (Sigma-Aldrich, catalog number: M2670)

7. Calcium chloride dihydrate (Sigma-Aldrich, catalog number: C7902)

8. Sucrose (Sigma-Aldrich, catalog number: S0389, CAS number: 57-50-1)

9. MEM (with Earl’s salts, without L-glutamine) (Gibco, catalog number: 21090-022)

10. HBSS 10× (without CaCl_2_/MgCl_2_) (Gibco, catalog number: 14185-045)

11. Horse serum, heat inactivated (Thermo Fisher Scientific, catalog number: 26050070)

12. Pen/Strep 10,000 units/mL (Gibco, catalog number: 15140-122)

13. B27 supplement (Gibco, catalog number: 17504-044)

14. Ara-C (Sigma-Aldrich, catalog number: C1768)

15. Ascorbic acid (Sigma-Aldrich, catalog number: A92902)

16. HEPES buffer (Gibco, catalog number: 15630-049)

17. Sterile water (Sigma-Aldrich, catalog number: W3500)

18. Lipofectamine 2000 (Invitrogen, catalog number: 11668030)

19. OptiMEM (Gibco, catalog number: 31985-062)

20. Paraformaldehyde solution (PFA), 4% in phosphate buffer solution (Thermo Fisher Scientific, catalog number: 15670799)

21. Phosphate buffered solution (PBS) (Sigma-Aldrich, catalog number: P4417)

22. Antibodies for neuronal staining (NeuN) (Abcam, catalog number: ab104224) and astrocyte labelling (GFAP) (Abcam, catalog number: ab134436)

23. Milli-Q water (prepared using a Milli-Q purification system available in the laboratory)

*Note: Reagents 11–14 and antibodies should be stored at -20 °C. We suggest aliquoting reagents to minimise freeze/thaw cycles. PFA stocks should be stored within a designated flammable storage cabinet and used inside a fume hood.*

**Solutions**

1. Sucrose-rich Ringer solution (see Recipes)

2. Culture medium (see Recipes)

3. DNA–Lipofectamine mixture (see Recipes)

4. Ringer solution (see Recipes)

**Recipes**

**1. Sucrose-rich Ringer solution (for hippocampal slicing)**

**Table:** 

Reagent	Molecular weight (g/mol)	Final concentration (mM)	Quantity (mg)
Sucrose	342.3	119.8	41,000
Sodium chloride	58.44	64	3,738
Potassium chloride	74.55	2.5	186
Calcium chloride dihydrate	147.02	0.5	73.5
Magnesium chloride hexahydrate	203.3	7	1,423
Sodium bicarbonate	84.01	25	2,100
Glucose	180.2	10	1,802
Sodium monophosphate	120	1.25	150
Total	-	-	1 L

*Reagents should be dissolved in 1 L of Milli-Q water; pH = 7.4; osmolarity ~300 mOsm/kg.*

*To avoid precipitation, the solution should be bubbled with 95% O_2_ and 5% CO_2_ before adding calcium chloride. To maintain sterility, decant into a 50 mL syringe fitted with a 0.22 μm filter. Pass the solution through the filter into a sterile container. We recommend always using a freshly prepared solution.*

**2. Culture medium**

**Table:** 

Reagent	Stock concentration	Final concentration	Volume (mL)
MEM (with Earl’s salts, without L-glutamine)	1×	0.50×	25
HBSS (without CaCl_2_/MgCl_2_)	10×	0.25×	1.25
Horse serum	100%	25% (v/v)	12.5
Pen/Strep	10,000 U/mL	100 U/mL	0.5
Glucose	1 M	16 mM	0.8
Ascorbic acid	250 mM	0.5 mM	0.1
HEPES buffer	1 M	8 mM	0.4
B27 supplement	50×	0.50×	0.5
Sterile water	-	-	8.95
Total	-	-	50 mL

*Note: Culture medium should be prepared under sterile conditions in a tissue culture hood. The medium can be stored at 4 °C for the duration of tissue maintenance.*

**3. DNA–Lipofectamine mixture (amount per one slice)**

**Table:** 

Reagent	Quantity or volume	
	DNA:Lipofectamine ratio 1:2	DNA:Lipofectamine ratio 1:1
DNA	2.5 μg	3.5 μg
Lipofectamine 2000	5 μL	3.5 μL
OptiMEM	up to 10 μL	up to 10 μL

*This recipe is for the transfection of a single organotypic hippocampal slice with the PIEZO1 sensor GenEPi. For further guidance, please see General note 3. In our experiments, the concentration of DNA ranged from 400 to 1,000 ng/μL; we used a mass of 2.5 or 3.5 μg.*

**4. Ringer solution [12]**

**Table:** 

Reagent	Molecular weight (g/mol)	Final concentration (mM)	Quantity (mg)
Sodium chloride	58.44	126	7,358
Potassium chloride	74.55	3	224
Calcium chloride dihydrate	147.02	1.25	150
Magnesium chloride hexahydrate	203.3	2	294
Sodium bicarbonate	84.01	2	493
Sodium monophosphate	120	26	2,184
Glucose	180.2	10	1,802
Total	-	-	1 L

*Reagents should be dissolved in 1 L of Milli-Q water; pH = 7.4; osmolarity ~300 mOsm/kg. Solutions can be stored at 4 °C for up to a week. To avoid precipitation, the solution should be bubbled with 95% O_2_ and 5% CO_2_ before adding calcium chloride.*

**Laboratory supplies**

1. Isoflurane (Abbvie, catalog number: B506, or Henry Schein, catalog number: 1182097, or any available)

2. Carbogen cylinder (95% O_2_ and 5% CO_2_ gas mixture)

3. Spray bottle with 70% ethanol (Fisher Scientific, catalog number: BP82031GAL)

4. Petri dish 100 mm × 15 mm (Corning, catalog number: 351029)

5. Fine forceps (Fine Science Tools, catalog number: 11412-11 or any similar fine forceps)

6. 6-well plate (Thermo Fisher Scientific, catalog number:140675)

7. Millicel membrane inserts (Millipore, catalog number: PICM0RG50)

8. Disposable Pasteur pipette (Kimblex, catalog number: DWK883350-0575)

9. 1.5 mL Eppendorf tubes (Sigma-Aldrich, catalog number: T9661)

10. Corning Falcon 50 mL tubes (Sigma-Aldrich, catalog number: CLS352070)

11. P20 pipette (Gilson, catalog number: F144056M)

12. P200 pipette (Gilson, catalog number: F144058M)

13. P1000 pipette (Gilson, catalog number: F144059M)

14. Sterile pipette tips 1,000 μL (Gilson, catalog number: F171703)

15. Sterile pipette tips 200 μL (Gilson, catalog number: F171503)

16. Sterile pipette tips 10 μL (Gilson, catalog number: F171203)

17. Plastic 50 mL syringe (Merck, catalog number: XX1105005)

18. 0.22 μm syringe filters (Millex, catalog number: SLMPR25SS)

## Equipment

**Dissection and preparation**

1. Anaesthesia induction chamber (VetEquip, US) or any other available

2. Standard scissors (Fine Science Tools, catalog number: 14002-12)

3. Coarse forceps (Fine Science Tools, catalog number: 11652-10)

4. Spring scissors (Fine Science Tools, catalog number: 15025-10)

5. Fine forceps (Fine Science Tools, catalog number: 11412-11)

6. Vibratome (Leica, model: VT1200 S)

7. Double-edge prep blades (AccuThrive, catalog number: AVBL-3002-0000)

8. Bucket or plastic box for ice (any available)

**Tissue culturing and transfection**

1. Tissue culture hood

2. Humidified incubator (set at 37 °C with 5% CO_2_)

3. Bead heater or water bath

**Electrophysiology and visualisation**

1. Two-photon upright microscope (Femtonics, Budapest), connected to a tuneable femtosecond pulsed laser (Insight X3, Spectra-Physics/Newport); alternatively, a confocal microscope (Leica, model: DM2500)

2. XLPlan N 25× water-immersion objective (NA 1.05, Olympus)

3. Camera (any equipped with the microscope)

4. PC, workstation

## Software and datasets

1. Imaging software (MES, Femtonics; LAS X, Leica, either requires a license)

2. ImageJ/Fiji (ImageJ 1.54f or g, or any other version)

3. Microsoft Excel (freely available)

4. GraphPad Prism 10

## Procedure

**A. Preparation and maintenance of hippocampal slices in vitro**

1. Prepare all media and dissection solutions according to the recipes provided above.

2. Set up the preparation and culture area and prepare the tools for dissection and slicing. Sterilise the working area and wipe all surfaces and tools with 70% ethanol to maintain aseptic conditions and reduce the risk of contamination during tissue dissection and slicing.

*Note: If required, sterilise the biosafety cabinet hood by UV exposure for 1 h before use.*

3. Dissect the brain and prepare hippocampal slices. In this step, proceed with sacrificing isoflurane-anaesthetised neonatal pups in accordance with the procedures approved by your institution and local ethics regulations (check the relevant local protocols in advance). Immediately after decapitation, remove the brain and carefully transfer it to a Petri dish (100 mm × 15 mm) containing oxygenated sucrose-rich Ringer solution. The dish should be kept on ice to ensure a well-chilled solution. Dissect out the olfactory bulbs and the cerebellum, then split the hemispheres. Slice the brain tissue to 350 μm thickness at 10 μm/s using a Leica vibratome in ice-cold sucrose-rich Ringer solution. Once cut, collect the hippocampal regions and promptly transfer these slices to room-temperature culture medium. Alternatively, hippocampi can be dissected out from each hemisphere by gently pulling out with a spatula as detailed previously [13,14] and sliced into 350-μm-thick slices.

*Notes:*

*1. We recommend using pups between postnatal (P) day 4 and 6 (up to P7 for mouse pups), as older tissue is generally less viable and more sensitive to manipulation, increasing the risk of compromised tissue quality.*

*2. To preserve tissue health during slicing, we keep the sucrose-rich Ringer solution ice-cold and ensure thorough oxygenation.*

4. Plate the hippocampal slices for in vitro culturing: Prepare 6-well plates containing 1.5 mL of culture medium per well and place a membrane insert into each well. Equilibrate the plates in an incubator set at 37 °C, with 5% CO_2_, before plating the slices. Under sterile conditions, transfer the slices onto the surface of the membrane inserts using a Pasteur pipette, either a glass or plastic pipette with a wide tip, as detailed in our previous protocol [14] and originally described by Stoppini et al. [15]. We typically plate three to four slices per membrane insert.

*Notes:*

*1. For transfer from the vibratome to the incubator, slices can be kept temporarily in a small Petri dish containing oxygenated, room-temperature culture medium.*

*2. Avoid leaving excess liquid on the slice. Remove any spare medium gently from around a tissue slice using a pipette with a small tip, taking care not to touch the tissue. For further guidance, see General notes 1 and 2.*

5. During the evening of day 4 in vitro (DIV 4), add 10 μL of stock concentration of Ara-C (500 μM) directly into the culture medium of each well. Replace the culture medium the next morning (DIV 5).

*Note: If the culture medium changes colour between days 1 and 4 post-plating, replace it to maintain tissue health. Otherwise, no additional medium exchange is required until DIV 5.*

6. Maintain the slices by regular medium exchange: Replace the culture medium every two to three days. For this, warm the culture medium to 37 °C using a bead heater or water bath for approximately 15 min. Remove the old medium using a P1000 pipette, placing the tip slightly beneath the membrane insert or briefly lifting the insert with tweezers if needed. Ensure that all medium is removed before adding 1.5 mL of fresh culture medium to each well.

*Note: Do not leave liquid on the surface of the membrane insert or on the tissue slices.*

**B. Lipofectamine-based transfection of the PIEZO1 sensor GenEPi**

1. For expression of large DNA vectors, perform Lipofectamine-based transfection between days 5 and 7 in vitro (DIV 5–7), as larger constructs may require a longer time window for detectable expression. Earlier transfection is therefore optimal, although the procedure can be delayed until DIV 10–11 if required, depending on experimental conditions.

*Note: In our experience with GenEPi, the sensor expression efficiency is generally low when transfection is performed between one and two weeks post-plating (DIV 8–14).*

2. Warm the culture medium and OptiMEM to 37 °C using a bead heater or water bath for approximately 15 min.

3. Bring the DNA plasmid and Lipofectamine 2000 reagent to room temperature for 5 min before mixing.

4. Transfer the DNA construct, Lipofectamine 2000 reagent, sterile 1 mL Eppendorf tubes, OptiMEM, and culture medium into the culture hood.

5. Replace the old culture medium with fresh medium as described in step A6.

6. In the Eppendorf tube, combine DNA and Lipofectamine 2000 at the desired ratio, either 1:2 or 1:1 DNA (μg) to Lipofectamine 2000 (μL). Mix well by gently pipetting the solution up and down 3 times; do not vortex.

*Notes:*

*1. In our experiments with GenEPi, we tested different DNA quantities, including 2.5 and 3.5 μg, with proportionally scaled Lipofectamine 2000 volumes (see Recipe 3).*

*2. All values should be scaled according to the number of slices transfected.*

7. Incubate the mixture at room temperature for 20 min to allow DNA–lipid complex formation.

*Note: Incubation time can be extended up to 30 min to support efficient complex formation for large DNA constructs.*

8. Slowly add prewarmed OptiMEM to the DNA–Lipofectamine mixture so that the final volume is 10 μL per slice.

*Notes:*

*1. If the DNA concentration is low, a final volume of 15 μL per slice may be used.*

*2. See General note 3 for additional information on the DNA–Lipofectamine 2000 mixture.*

**Figure 1.**
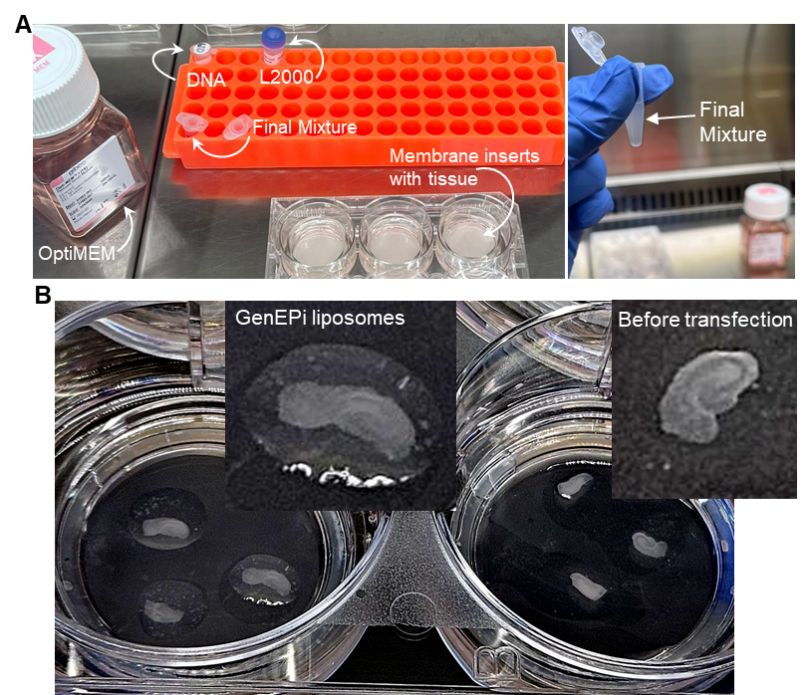
Preparation of DNA–Lipofectamine 2000 complexes and surface transfection of organotypic hippocampal slices. (A) The left image highlights the key reagents used, and the right image shows the DNA–Lipofectamine 2000 mixture ready for slice transfection. (B) Images of organotypic hippocampal slices (7 days in vitro, DIV) immediately after (left) or prior to (right) transfection. Note the GenEPi–liposome droplets directly on the surface of the tissue.

9. Mix the solution by slowly pipetting up and down three times using a P1000 pipette.

10. Incubate the diluted mixture at room temperature for 5 min to allow equilibration of the complexes.

11. Once all reagents are ready, bring the 6-well plate containing the tissue slices from the incubator (Figure 1). Using a pipette, apply a 10 μL droplet of the DNA–Lipofectamine mixture directly onto the surface of each slice. The droplet should visibly cover the slice without spreading across the membrane. Repeat for all slices.

*Note: Avoid touching the tissue with the pipette tip and ensure the droplet remains intact on the slice surface, as mechanical disturbance compromises tissue integrity and can reduce transfection efficiency.*

12. Return the 6-well plate to the incubator. Proceed with all remaining plates.

*Note: Handle the plates carefully to minimise movement and avoid disturbing the transfection droplets.*

13. Change the culture medium every 2–3 days post-transfection, without lifting the membrane inserts from the wells or disturbing the tissue.

*Note: We recommend the first medium change at 3 days post-transfection to allow sufficient time for liposome diffusion and cellular uptake. However, if the medium becomes very yellow, it should be changed as early as 2 days after transfection to preserve tissue viability.*

**C. Detecting PIEZO1 sensor expression in pyramidal neurons**

1. Maintain organotypic hippocampal slices in an incubator until use, replacing the culture medium every 2–3 days to preserve tissue health and support sustained expression of the PIEZO1 sensor GenEPi.

2. Monitor expression over time by systematically tracing fluorescence intensity across hippocampal regions. The earliest detectable GenEPi signal in pyramidal neurons can be observed around 1 week post transfection, with expression increasing at later time points (e.g., 2–3 weeks post transfection).

3. For quantitative assessment, use consistent imaging settings and exposure times across experimental groups (e.g., different time points or DNA amounts) to allow reliable comparison of expression dynamics.

4. Depending on the experimental aims, proceed with the tissue for either live imaging, functional assays, or fixation for post hoc staining. For live imaging, maintain the slices on the microscope stage under physiological conditions (e.g., perfusion with oxygenated Ringer solution).

5. To evaluate GenEPi transfection level and the spatial distribution of the PIEZO1 sensor across hippocampal neurons over time, fix organotypic slices at different time points (1–3 weeks post transfection) in 4% PFA for 1–1.5 h at room temperature, followed by extensive washes with PBS.

*Notes:*

*1. PFA is toxic and teratogenic; therefore, fixation should be performed inside a fume hood with appropriate care and waste handling.*

*2. We fixed tissue directly onto the membrane inserts by submerging the membrane in 1 mL of 4% PFA in 6-well plates. After fixation, a minimum of five 1-mL PBS washes is recommended.*

6. For fixed tissue, acquire Z stack images to visualise GenEPi expression in pyramidal neurons across different depths. Two-photon excitation (2PE) microscopy was used for imaging. To optimise sensor excitation, images were acquired at 910 nm, with laser intensity adjusted to minimise photobleaching. Laser power under the objective was always kept below 5–7 mW; frame resolution was set to 512 × 512 pixels, and Z-stack typically comprised 20–40 optical sections collected at 2.0-μm step intervals. Imaging was consistently initiated from the top surface of each slice to ensure standardised sampling depth and minimise variability between sections. Alternatively, confocal imaging can be used. Imaging protocols should be standardised across all tissue samples, including consistent imaging parameters, to ensure valid quantification between experimental groups.

*Note: To improve comparability across samples, we recommend that the location of regions of interest selected for imaging across samples should also be standardised.*

7. Fixed slices can be immunostained for cell-type markers (e.g., NeuN for neurons, GFAP for astrocytes) and imaged to confirm GenEPi expression across brain cell types.

## Data analysis

All generated Z-stacks were exported as TIFF files containing sequential focal planes through the depth of the tissue region.

*Note: Ensure that all TIFF images are calibrated before starting the analysis.*

**A. Creating Z-projection images of pyramidal neurons**

1. Open the Z-stack file in ImageJ/Fiji software.

2. Go to *Image* > *Color* > *Channels Tool*. When the pop-up window appears, select *More > Green*.

*Note: Other display colours can be used; we routinely use green for these images.*

3. Go to *Image > Adjust > Brightness/Contrast > Auto*.

*Note: Nonlinear adjustments to brightness and contrast should not be applied, as these may alter consistency across samples.*

4. Starting at the tissue surface, use the scroll bar located at the bottom of the Z-stack to move through the focal planes and identify the first plane showing clear neuronal expression of the sensor.

**Figure 2.**
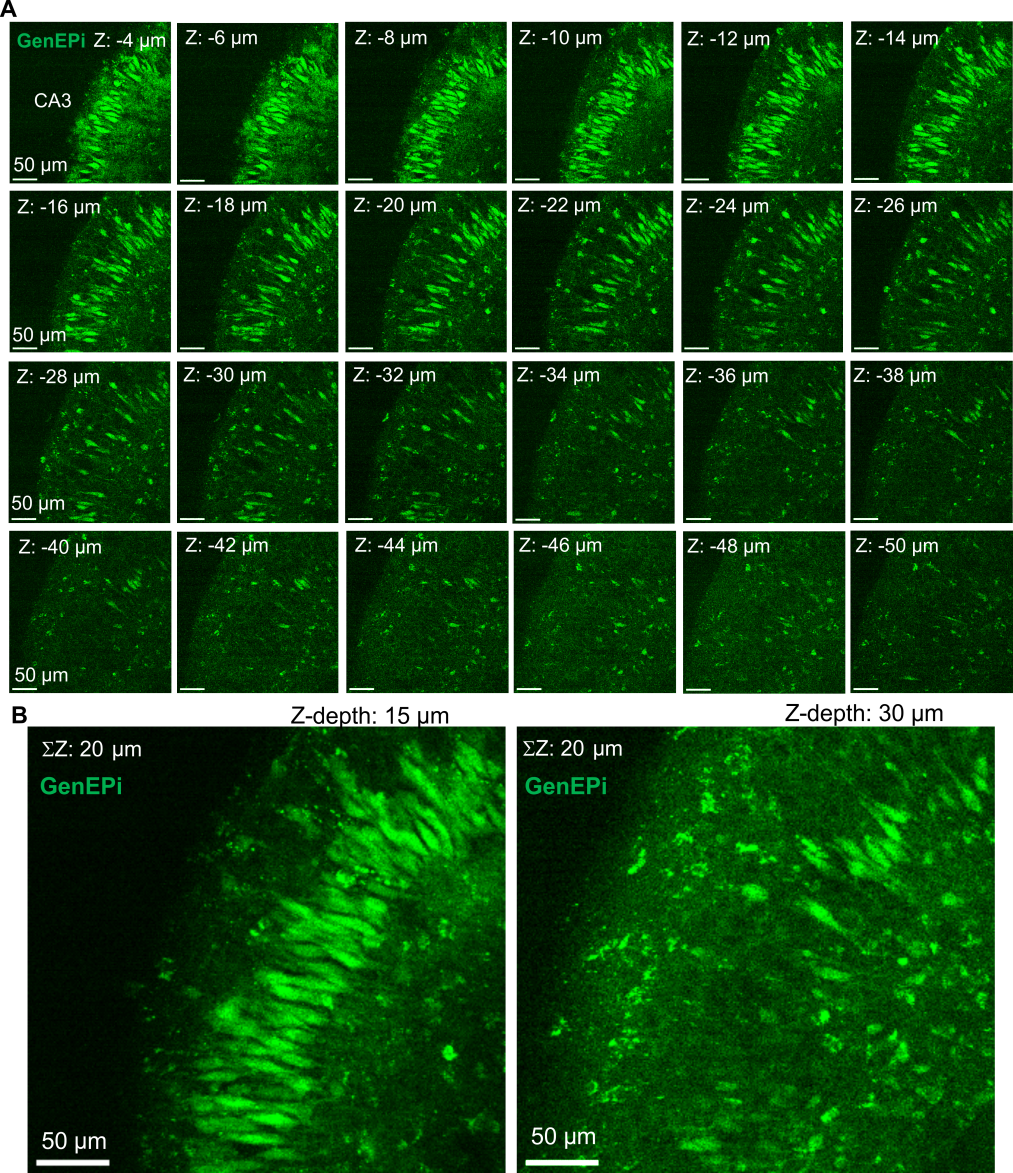
Creating Z-projection images for visualising pyramidal neurons expressing GenEPi across a hippocampal slice. (A) Representative images of individual sequential focal planes from the original Z-stack file, spanning tissue depths to above 50 μm, until GenEPi expression can be visualised. The images demonstrate robust GenEPi expression in CA3 pyramidal neurons. (B) Representative Z-projection images generated from the above individual Z-planes, displaying GenEPi-expressing CA3 pyramidal neurons. All representative images display slices transfected with GenEPi (3.5 μg) at 2 weeks post-transfection.

*Note: For clarity, we present a representative Z-stack as a sequence of single focal plane images (Figure 2A).*

5. Create a Z-projection image by selecting *Image > Stack > Z Project*. Enter the first and last focal plane numbers and select *Sum Slices* from the drop-down menu.

*Notes:*

*1. The Z-projection image should be large enough to reconstruct individual neurons accurately while avoiding overlap between neurons in adjacent planes.*

*2. Under our imaging conditions and organotypic preparation, Z-stacks of ~40–45 μm were typically collected.*

*3. In our preparation, individual pyramidal neurons are ~10–12 μm thick; therefore, we generated Z-projection images of up to ~22 μm to sufficiently capture as many neurons as possible within the pyramidal layer.*

6. Repeat step A5 to generate a series of Z-projection images until no further sensor signal is detected through the tissue depth (Figure 2B). Leave a suitable interval between successive focal planes to avoid including the same neurons in more than one projection.

*Note: We kept an interval of 5–10 μm between successive focal planes, which was sufficient to prevent overlap of sampled neurons under our imaging conditions.*

7. Apply the same colour and brightness/contrast settings to all Z-projection images by repeating steps A2–3 for each image (Figure 2B).

*Note: Saving the processed Z-projection images as TIFF files for later use is recommended.*

**B. Analysis of PIEZO1 sensor expression at the single-neuron level**

**Figure 3.**
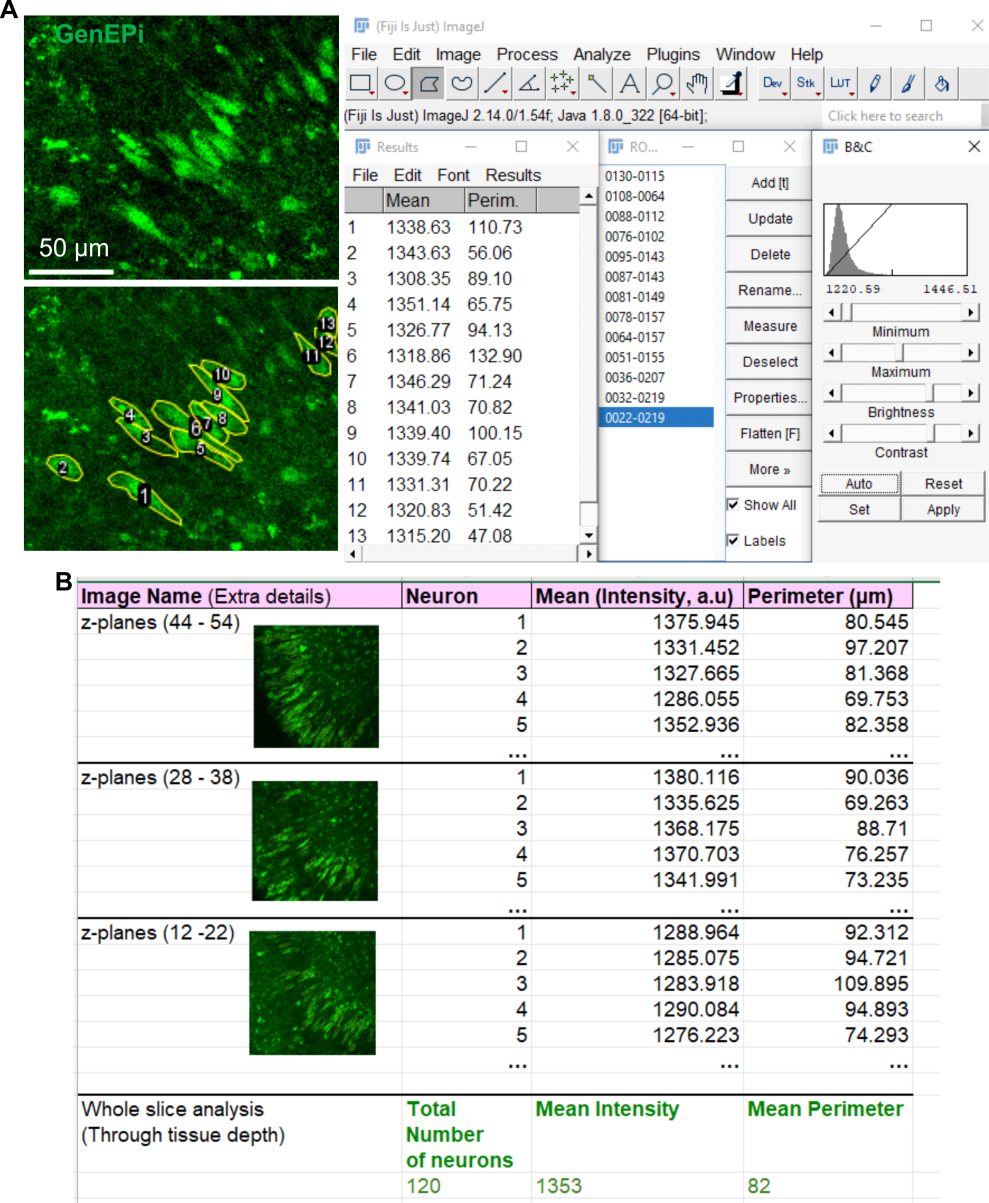
ImageJ image analysis workflow for GenEPi-expressing pyramidal neurons. (A) Left panel: Representative Z-projection image (from Figure 2B) at high magnification, showing GenEPi-expressing pyramidal neurons (upper) used to outline individual neurons that show a clear, homogeneous GenEPi signal pattern (lower) for quantitative analysis. The table on the right shows the measurement results (mean fluorescence intensity and perimeter) for the outlined neurons. (B) Example Excel spreadsheet for analysis of obtained measurements. All representative images display slices transfected with GenEPi (3.5 μg) at 2 weeks post-transfection (~21 DIV).

1. Open a Z-projection image derived from the original TIFF file generated in the previous steps.

2. In the ImageJ toolbar, select the *Polygon Selection* tool.

3. Manually outline a GenEPi-expressing neuron using this tool (Figure 3).

4. Right-click the outline and select *Add to ROI Manager*. This will open a window in which the current region of interest (ROI) is saved.

**Figure 4.**
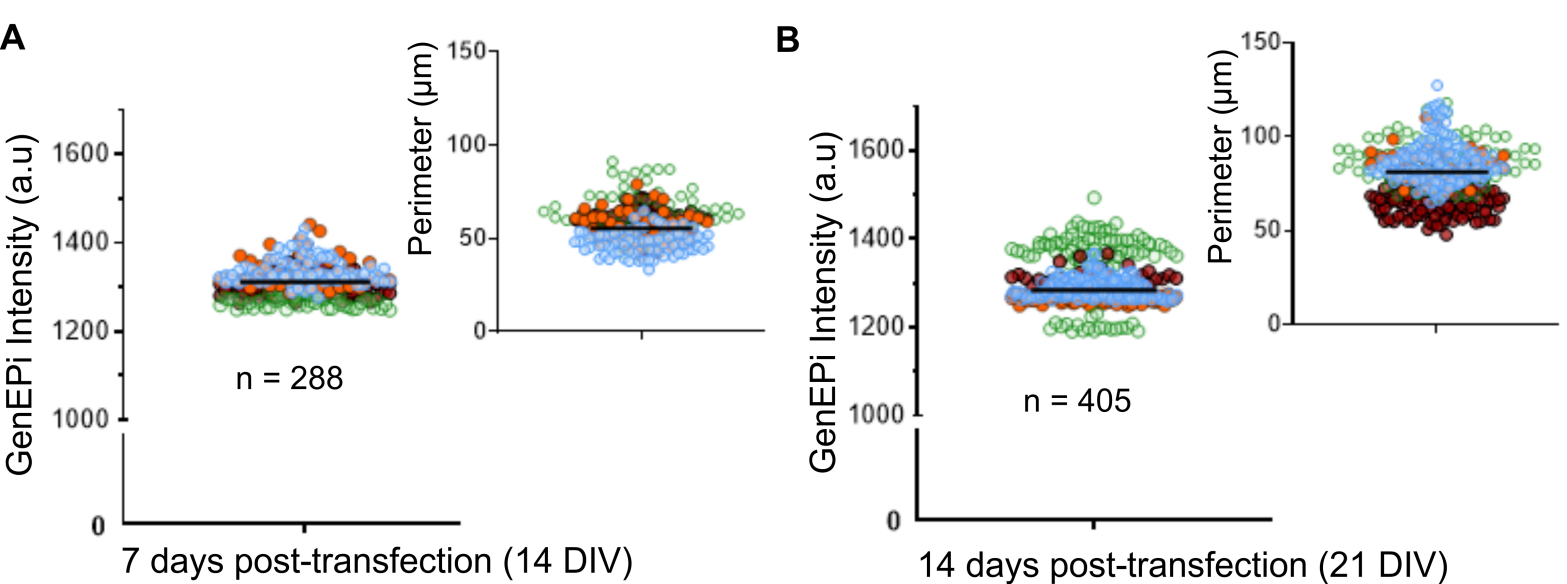
GenEPi expression in pyramidal neurons at 7 days (left plots) and 14 days (right plots) post-transfection in organotypic hippocampal slices. Scatter plots display the absolute fluorescence intensity of GenEPi and the perimeter of individual pyramidal neurons (pooled CA3 and CA1). The total number of pyramidal neurons analysed is indicated, with data collected from 4 independent slices (distinguished by colour). Lines indicate median values for non-parametrically distributed datasets.

*Note: Selecting* Show all *and* Labels *allows all ROIs to be viewed on the Z-projection (see Figure 4). This helps avoid re-selecting the same neuron and facilitates identifying cells (ROIs) in the results table in later steps.*

5. Repeat steps B3–4 for all GenEPi-expressing neurons within the field of view.

*Notes:*

*1. Our criteria for selecting pyramidal CA1 and CA3 neurons were to include (i) only neurons within the pyramidal layer, which (ii) show a homogeneous signal across the cell membrane.*

*2. Transmitted light images can help to identify neuronal boundaries more clearly.*

**Figure 5.**
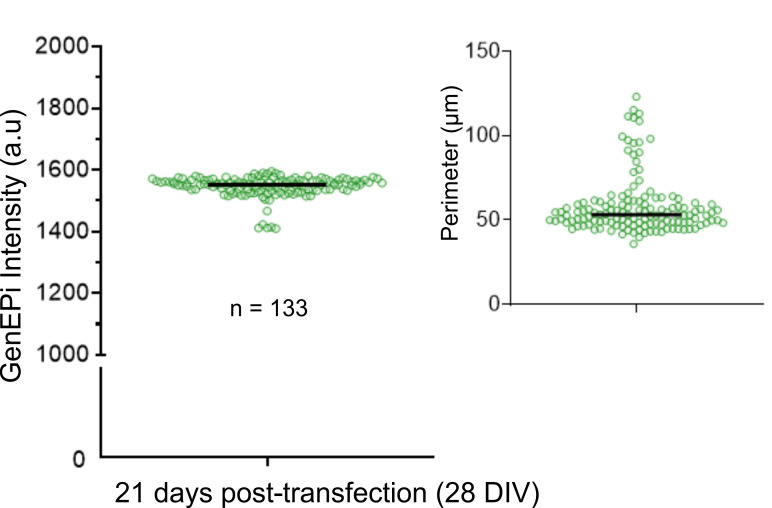
Lipofectamine-based delivery of GenEPi in organotypic hippocampal slices results in stable expression in pyramidal neurons for 3 weeks post-transfection. Scatterplots display the absolute fluorescence intensity of GenEPi and the perimeter of individual pyramidal neurons (pooled CA3 and CA1). Lines indicate median values for non-parametrically distributed datasets. The total number of pyramidal neurons analysed is indicated, with data collected from one slice preparation.

6. In the toolbar, select *Analyse > Set Measurements* and check the parameters for analysis, such as *Mean grey value, Perimeter*, and others. Then, click *OK*.

7. To extract measurements for all ROIs, open the ROI manager, select *More > Multi Measure*, leave the additional options unchecked, and click *OK*. This generates a results table with the selected parameters for each ROI, representing individual neurons.

8. Copy the values from the results table and paste them into an Excel spreadsheet or other software used for analysis (Figure 3B).

9. Save all Z-projection images with their ROIs overlaid for future reference.

10. Repeat steps B1–9 for each Z-projection image derived from the original Z-stack file.

11. Combine data from all Z-projection images obtained from the same Z-stack file into a single dataset representing that image. Using Excel or other software, generate summary statistics for the selected parameters (Figures 4 and 5).

*Note: Additional analysis can be performed using ImageJ, Excel, and a statistical package of choice.*

## Validation of protocol

To validate this protocol, we expressed the large plasmid GenEPi (12,912 bp), a genetically encoded fluorescent sensor of PIEZO1, in organotypic hippocampal slices. Slices were transfected at 7 DIV with 2.5 μg of GenEPi by applying a 10 μL droplet of DNA–Lipofectamine 2000 mixture directly to the tissue surface. Tissue was subsequently fixed at 7, 14, and 21 days post-transfection. We then performed 2PE imaging to quantify GenEPi expression in CA1 and CA3 pyramidal neurons by counting the number of GenEPi-expressing neurons and measuring signal intensity in individual neurons. Through this approach, we demonstrate that the protocol results in robust GenEPi expression in hippocampal pyramidal neurons. For the analysis, we focused on CA1 and CA3 pyramidal neurons, as these glutamatergic neuronal populations are most vulnerable to suboptimal conditions and therefore serve as sensitive indicators of tissue health [16–18].

Our findings demonstrate a successful vector transfection in pyramidal neurons, with detectable GenEPi signal observed as early as 7 days post-transfection in organotypic hippocampal slices (Figure 4). We counted 288 pyramidal neurons with detectable GenEPi signal, whose intensity ranged from 1,248 to 1,440 a.u. (*p* < 0.001, Shapiro-Wilk test; *p* < 0.05, Lilliefors test; Figure 4). These data provide evidence that the protocol is suitable for early experimental applications. From 7 to 14 days post-transfection, the number of GenEPi-expressing neurons increased to 405, while fluorescence intensity remained at a similarly high level, ranging from 1,190 to 1,490 a.u. (*p* < 0.001, Shapiro-Wilk test; *p* < 0.05, Lilliefors test; Figure 4). Together, these findings indicate sustained and reliable expression of the PIEZO1 sensor GenEPi in pyramidal neurons over time.

To further assess the long-term effects of transfection, slices were maintained for 3–4 weeks post-transfection, corresponding to 26–33 DIV. Importantly, detectable GenEPi expression was observed at later time points in 133 neurons, with signal intensity ranging from 1,409 to 1,594 a.u. (*p* < 0.001, Shapiro-Wilk test; *p* < 0.05, Lilliefors test; Figure 5). These results demonstrate that the protocol supports sustained expression and is compatible with longer-term experimental applications without compromising the GenEPi signal.

**Figure 6.**
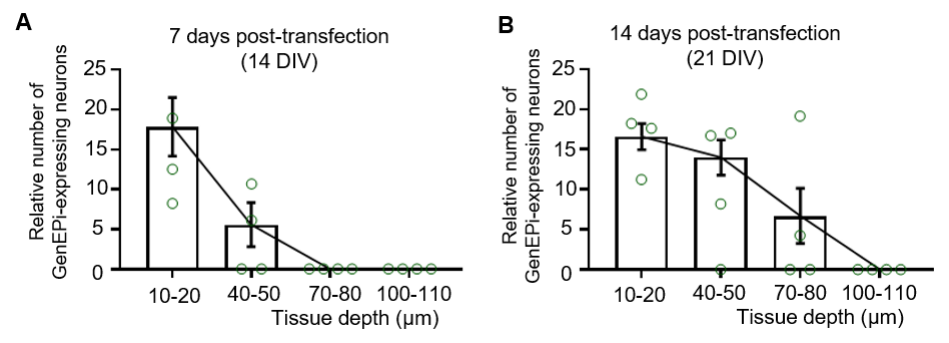
Relative number of GenEPi-expressing neurons across the tissue depth of organotypic hippocampal slices at (A) 7 days and (B) 14 days post-transfection. Bar charts display the mean ± SEM of the total number of GenEPi-expressing cells, relative to an arbitrarily defined area of 200 μm^2^, in hippocampal slices transfected with 2.5 μg of GenEPi (n = 4 per time point).

In addition to quantifying the number of GenEPi-expressing pyramidal neurons and signal intensity, we assessed transfection efficiency by quantifying GenEPi-expressing neurons across tissue depth. At both 7 and 14 days post-transfection, the number of GenEPi-expressing neurons was highest at the tissue surface (Figures 2A and 6). This is consistent with the direct application of the DNA–Lipofectamine complex onto the tissue surface. The number of GenEPi-expressing neurons became sparser and more dispersed with increasing depth (Figure 2A), reaching up to 100 μm (Figure 6A). Notably, the number of GenEPi-expressing cells increased in slices maintained for longer periods, and GenEPi expression extended more extensively into deeper tissue layers (Figure 6). This suggests that expression and/or liposome penetration improves over time.

Collectively, these findings demonstrate that the protocol enables robust and spatially appropriate expression at the tissue surface, making it well-suited for standard imaging configurations and experimental interventions. This approach contrasts with conventional cell culture approaches, in which DNA–Lipofectamine complexes are applied in bulk to the culture medium for short incubation periods [1,11]. The next steps will be to test the sensor functionality in live-cell imaging, similar to our previous studies of other sensors, such as those monitoring neurotransmitter dynamics of glutamate [18] and GABA [19].

## General notes and troubleshooting

**General notes**

1. Organotypic hippocampal slices should be maintained for at least 5 days prior to transfection. In our preparation, hippocampal slices were cut to a thickness of 350 μm to help preserve tissue integrity.

2. To maximise yield and account for variability in tissue quality, we recommend using rat pups for slice preparation. Although the method can be performed with mouse tissue, the smaller size of the hippocampus makes the preparation more challenging, and the soft brain tissue is more vulnerable and prone to damage.

3. The DNA–Lipofectamine 2000 mixture should be optimised for the specific DNA construct. We recommend a starting ratio of 1 μg DNA to 2 μL Lipofectamine 2000, adjusting the final volume to 10 μL with OptiMEM. To achieve this small volume, use a high-concentration DNA whenever possible. If this is not feasible, the final volume may be extended to 15 μL; however, larger droplet volumes are less stable on the slice surface, as the droplet tends to spread or move, leading to uneven expression. Depending on the size and stability of the DNA construct, further optimisation of the DNA:Lipofectamine ratio may be required. Ratios of 1:1 and 1:2 are recommended for initial testing.

4. Despite maintaining cultures for up to 3–4 weeks post-transfection and using two-photon microscopy, we did not detect substantial expression at a tissue depth below ~100 μm, suggesting a depth-limited penetration of DNA–Lipofectamine complexes.

5. Variability between and within preparations is expected and may arise from various factors, e.g., tissue age, slice health, etc. To account for this, testing several tissue preparations is required.

6. Although demonstrated in organotypic hippocampal slices, this approach can also be applied to other brain regions.

7. Achieving robust expression at the tissue surface is particularly important, as imaging is performed from the top of the slice; imaging from the underside is not feasible due to high autofluorescence from the culture membrane.

**Troubleshooting**

**Problem 1:** Low transfection efficiency.

Possible causes: The DNA amount is too low, the liposome diffusion time is insufficient, or the DNA–Lipofectamine 2000 ratio is suboptimal.

Solutions: Optimise the transfection conditions for the specific DNA construct. Test different DNA amounts (e.g., 0.5–5 μg of DNA per slice). Also, vary the DNA–Lipofectamine 2000 ratios (see General note 3). Additionally, extend the culture time after transfection.

**Problem 2:** Expression restricted to the tissue surface.

Possible cause: Limited penetration of liposomes through the cell layers.

Solutions: Extend the culture time to allow for higher expression across depths. When applying the DNA–Lipofectamine droplet to the slice, ensure that it remains stable on the surface without spreading or moving, as it can lead to uneven expression.

**Problem 3:** High expression variability between slices.

Possible causes: Differences in slice health or thickness, or variation in imaging protocol.

Solutions: Analyse multiple regions per slice but maintain the same imaging protocol settings. When applying the transfection droplet, ensure that it remains centred and undisturbed to reduce subregion variability.
